# Supplementary material for: Universal perpendicular orientation of block copolymer microdomains using a filtered plasma
Source: Nat Commun. 2019 Jul 2;10:2912. doi: 10.1038/s41467-019-10907-5 (PMC6606568; doi:10.1038/s41467-019-10907-5)
Supplement: Supplementary file 1 — Supplementary Information [file 41467_2019_10907_MOESM1_ESM.pdf]

Supplementary Information – Oh et al.

Universal Perpendicular Orientation of Block Copolymer Microdomains using a Filtered Plasma

Jinwoo Oh[1,2], *Hyo Seon Suh*[3], *Youngpyo Ko*[1], *Yoonseo Nah*[1], *Jong-Chan Lee*[2], *Bongjun Yeom*[4], *Kookheon Char*[2], *Caroline A. Ross*[5], *Jeong Gon Son*[1,6]*

[1]Photo-Electronic Hybrids Research Center, Korea Institute of Science and Technology, Seoul, 02792, Republic of Korea

[2]School of Chemical & Biological Engineering, Seoul National University, Seoul, 08826, Republic of Korea

[3]imec, Kapeldreef 75, 3001 Leuven, Belgium

[4]Department of Chemical Engineering, Hanyang University, Seoul, 04763, Republic of Korea

[5]Department of Materials Science and Engineering, Massachusetts Institute of Technology, Cambridge, Massachusetts, 02139, USA

[6]Division of Energy & Environment Technology, KIST School, Korea University of Science and Technology, Seoul, 02792, Republic of Korea

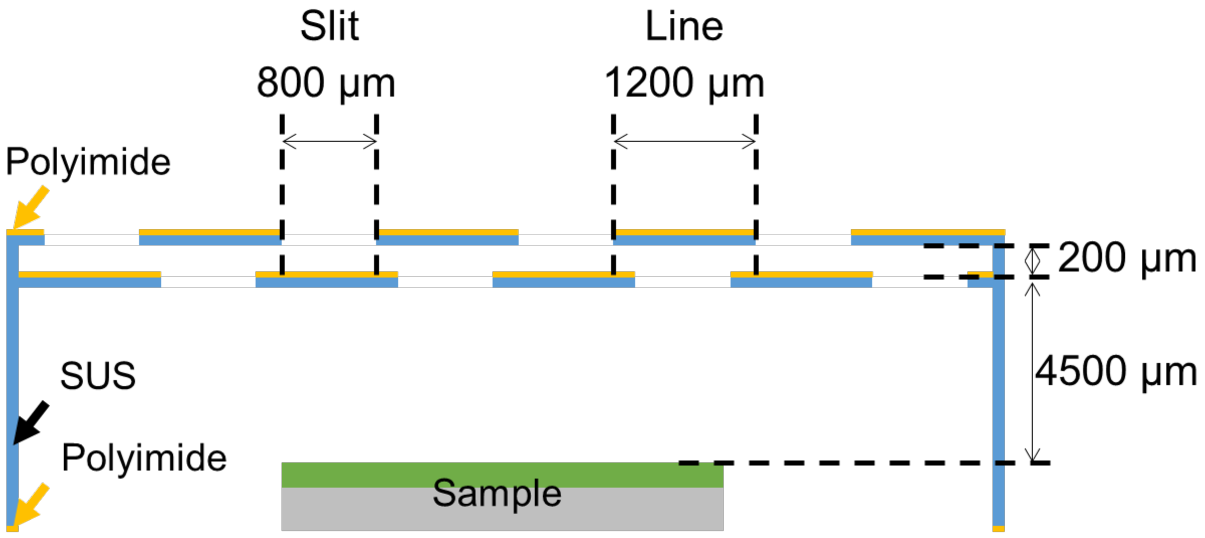

Supplementary Figure 1. Schematic image of the staggered filter. The staggered bilayer filter with 200 µm gaps consisted of periodic 800 µm wide slits and 1,200 µm width opaque regions to block the VUV/UV photons and accelerated ions. The filter is made of stainless steel and wrapped with polyimide tape for insulation. Masks without polyimide coating also showed the same results.

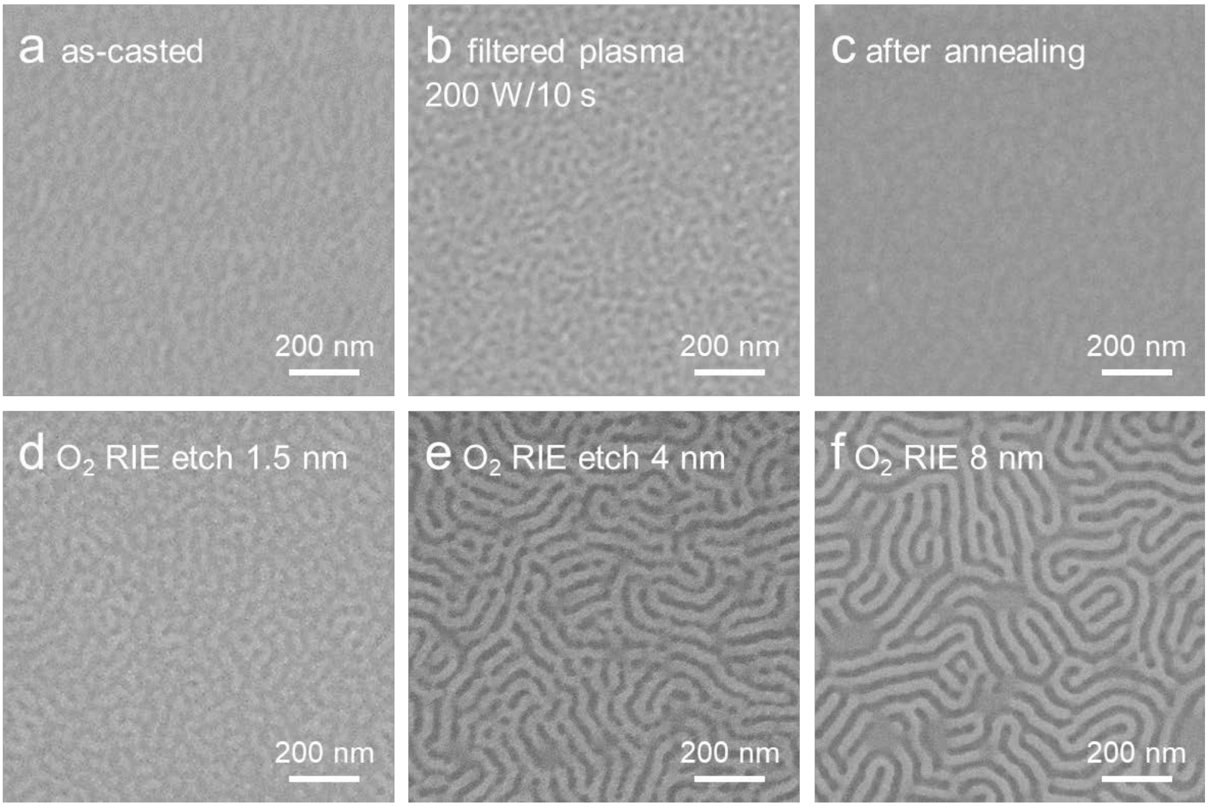

Supplementary Figure 2. Surface morphologies of SML100 thin film after the filtered plasma process. SEM top surface morphologies of (a) as-cast film, (b) after filtered plasma treatment (200 W, 10 s), (c) after filtered plasma treatment, then thermal annealing at 220 °C for 4 h, followed by O_2 reactive ion etching to etch (d) 1.5 nm, (e) 4 nm and (f) 8 nm of the top surface layer and reveal the underlying perpendicular lamellae.

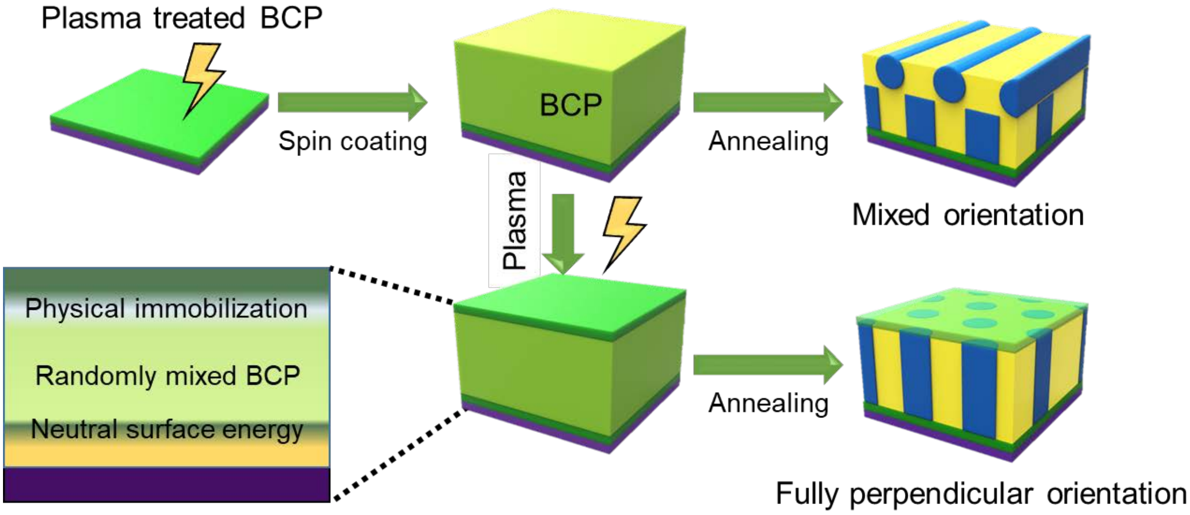

Supplementary Figure 3. Schematic images of the bottom plasma (BP) treatment and the sandwich plasma (SWP) treatment. When the plasma treatment with the filter is performed to cross-link and physically immobilize the surface of the BCP film, the cross-linked layer not only provides a neutral top surface to the underlying BCP film, but also provides a neutral bottom interface when a new BCP film is introduced onto the crosslinked layer. Thin (~7 nm) BCP films with our filtered plasma treatment (BP) can act like a random copolymer brush that forms a neutral interface on the substrate so that the perpendicular orientation from the bottom interfaces can be formed. When the BP and TP conditions are performed in the same sample, fully perpendicular oriented BCP films can be produced.

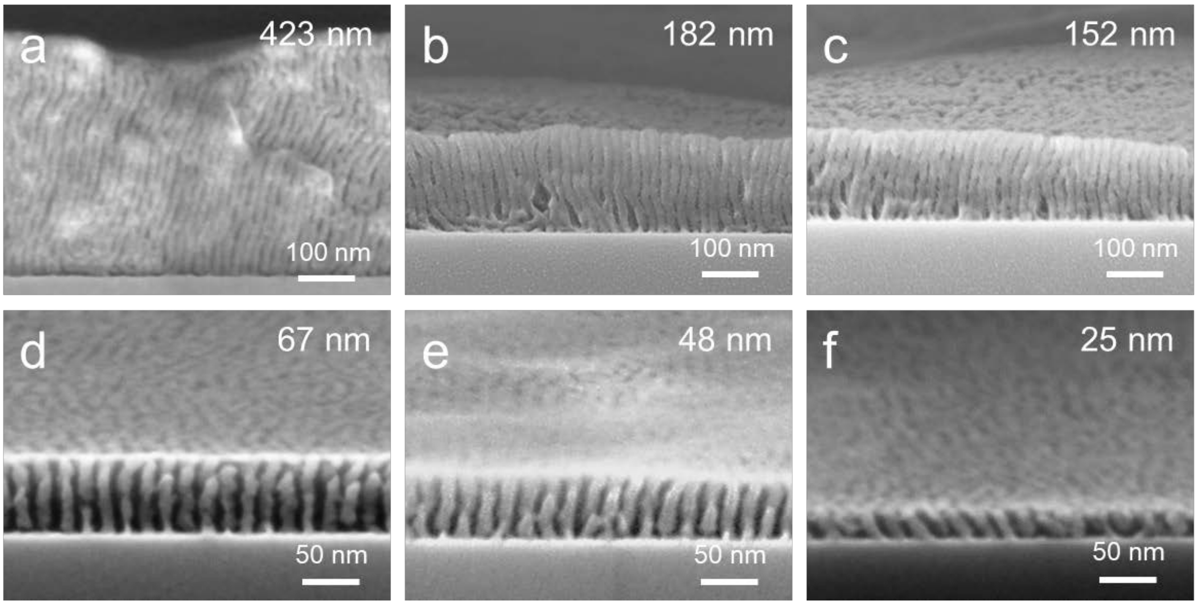

Supplementary Figure 4. Orientation of sandwich-plasma treated SDC16 films with different thickness. Cross-sectional SEM image of SDC16 with different film thickness of (a) 423 nm, (b) 182 nm, (c) 152 nm, (d) 67 nm, (e) 48 nm and (f) 25 nm.

Supplementary Note 1. GISAXS analysis of filtered plasma BCP films.

Supplementary Figure 5 shows GISAXS patterns from the SDC16 films with BP, TP and SWP condition at 0.08° ($\alpha < \alpha_c$) and 0.14° ($\alpha > \alpha_c$) to observe the orientation of the BCP microdomains at the surface or through the film.[1] GISAXS patterns at the surface of the SDC16 film with the BP condition (Supplementary Figure 5a and d) at 0.08° show strong hexagonal (11) diffraction peaks of parallel oriented cylinders at q_x = 0.025 Å^{-1} and a very faint (10) peak at q_x = 0.030 Å^{-1}, which indicates dominant parallel orientation of cylinders at the top surface. At 0.14° which is sensitive to the inside of BP-SDC16 films, the scattering pattern shows strong perpendicular (10) peak with a weak parallel oriented hexagonal (11) peak indicating that a perpendicular structure mainly exists within the film. On the other hand, at the surface (0.08°) of SDC16 film with TP condition (Supplementary Figure 5b and e), only strong peaks at q_x = 0.030 Å^{-1} from the perpendicular oriented cylinder at the top surface are observed. At higher incidence angle (0.14°), GISAXS patterns of SDC16 film with TP shows both (11) and (10) peaks from the parallel and perpendicular orientation of cylinders in the films. The SDC16 film with SWP condition (Supplementary Figure 5c and f) shows only a strong (10) peak at q_x = 0.030 Å^{-1} from the perpendicular oriented cylinders at 0.08° and 0.14° incidence angles, and indicates fully perpendicularly oriented cylinders in the films with 20.9 nm period.

GISAXS patterns from the SML100 films with BP, TP and SWP condition are shown in Supplementary Figure 6.[2] Since PS-*b*-PMMA has almost the same surface energy between the two domains at 220 °C, peaks along the q_x axis (~0.0117 Å^{-1}) related to the perpendicularly oriented lamellae with ~54 nm full pitch on the surface are commonly observed in BP, TP and SWP. However, by analyzing the scattering density around the q_z axis (Supplementary Figure 6d-f), information on parallel orientation can be obtained from the presence of peaks. In the case of BP, peaks along the q_z axis are clearly observed at 0.08° and 0.14°, which means that

the parallel orientation was formed at the film surface. However, in the case of TP, no peak on the q_z axis was confirmed on the surface (0.08°), but the peaks for the parallel orientation were observed inside of the film (0.14°). In the case of SWP, it was difficult to observe the parallel orientation peaks both at the surface and inside the film. The shape of the scattering pattern in which the peaks appearing in the truncated vertical lamellae are diffused upward can also indicate whether perpendicular orientation is present. This is also observed only on the surface of TP and SWP films, and it can be seen that the perpendicular orientation is well formed through the plasma with the filter approach.

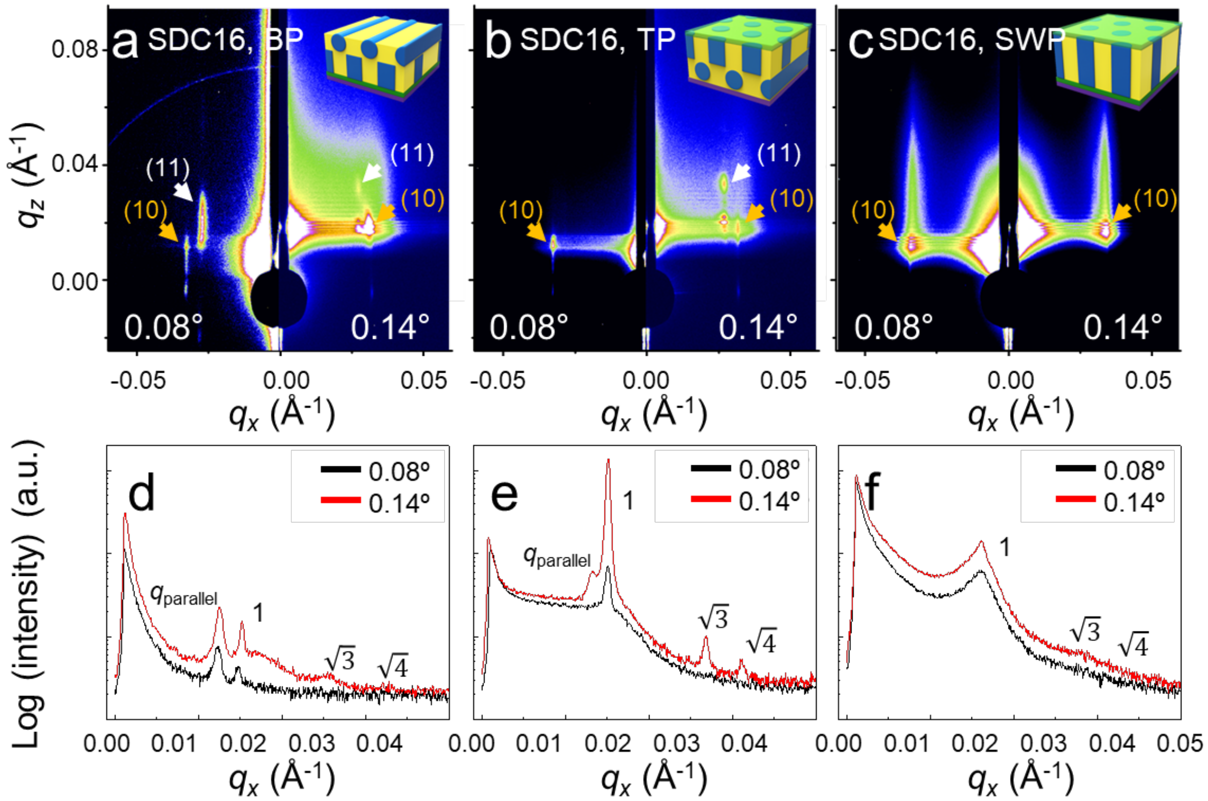

Supplementary Figure 5. GISAXS patterns of PS-*b*-PDMS (SDC16) with different plasma treated geometries. Scattering patterns of (a) BP, (b) TP and (c) SWP conditions of SDC16 films with different incidence angles of 0.08° (below the critical angle, α_c) to 0.14° (above the α_c) to obtain orientation information only from the surface or through the whole film. (d-f) Extracted 1D scattering intensity peaks at the Yoneda line of the GISAXS patterns of (d) BP, (e) TP, and (f) SWP condition SDC16 films with different incidence angles of 0.08° (black) to 0.14° (red).

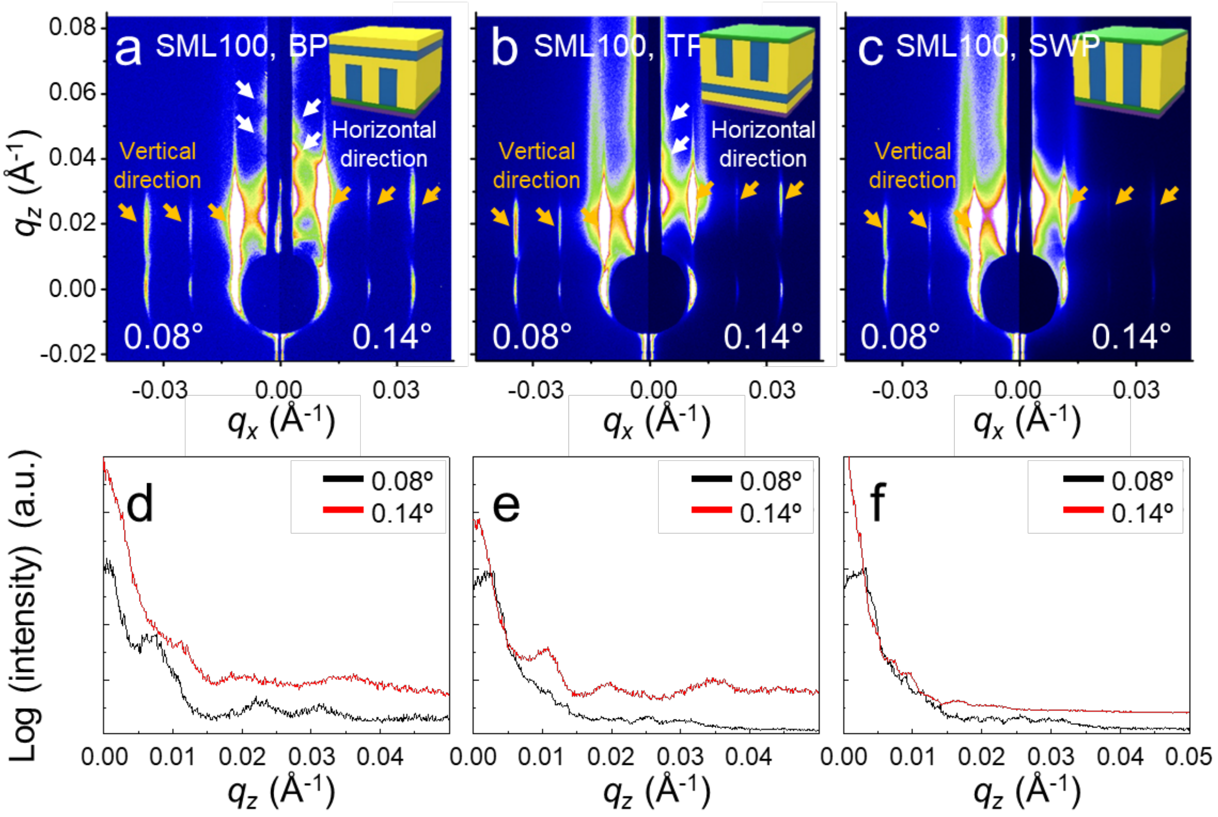

Supplementary Figure 6. GISAXS patterns of PS-*b*-PMMA (SML100) with different plasma treated geometries. Scattering patterns of (a) BP, (b) TP and (c) SWP conditions of SML100 films with different incidence angles of 0.08° (below the critical angle, α_c) to 0.14° (above the α_c) to obtain orientation information only from the surface or through the whole film. (d-f) Extracted 1D scattering intensity peaks along the q_z line beside the beam stopper (-0.008 Å^{-1} < q_x < Å^{-1}) of the GISAXS patterns of (d) BP, (e) TP, and (f) SWP condition SML100 films with different incidence angles of 0.08° (black) to 0.14° (red).

Supplementary Note 2. Details of ARXPS and water contact angle measurements

In the C1*s* peak of the untreated PS film in Figure 4b, a small delocalized aromatic peak is present at 290.9 eV and a symmetrical large C-C peak is present at 284.1 eV. When the film is plasma treated without the filter for 10 s, the aromatic peak disappears and a C-O peak appears at 286.0 eV, but treatment with the filtered plasma for 60 s produced no peak change even at a 70° measurement angle which is more sensitive to the surface. In the case of UV-sensitive PMMA, Figure 4c, the O1*s* peaks of the ester (C-O, 533.2 eV and C=O, 531.6 eV) are reduced in the plasma-treated film, whereas measurements at 0° and even at 70° incidence angles show no change in the peaks for the filtered plasma treatment.

In the full-scan XPS spectra for the PS film after Ar plasma treatment without the filter in Supplementary Figure 15, fluorine and chloride not used in the experiment were detected at the film surface. This contamination may originate from previous use of these materials in the reactor and is undesirable. In contrast, the PS film after Ar plasma treatment with the filter did not exhibit any chemical contamination because the filter blocks not only UV but also accelerated ions from striking the film surface.

Water contact angle measurements of homopolymers, SML100, SDL43 and SVL84 films are compared in Figure 4d-f. When the films were treated with a filtered plasma, the contact angles (72.5° for SML100, 101.3° for SDL43, 70.7° for SVL84) were almost the same as the contact angles of pristine BCP films (72.7° for SML100, 102.6° for SDL43, 76.0° for SVL84) and had values intermediate between the contact angles of the respective homopolymers. However, the contact angles of the BCP films plasma-treated without the filter (40.3° for SML100, 76.0° for SDL43, 39.5° for SVL84) were much lower.

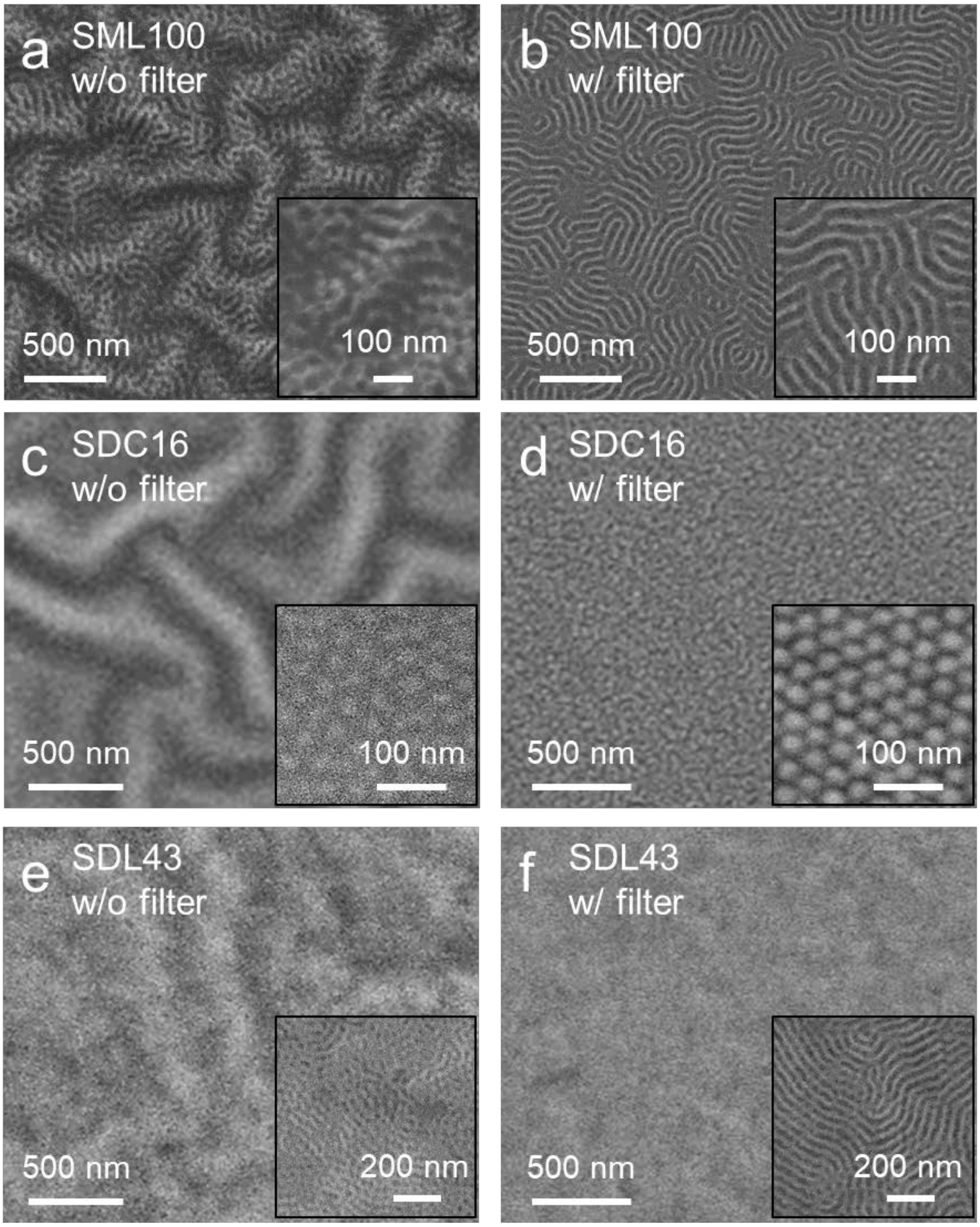

Supplementary Figure 7. Different morphologies between the plasma with and without filter. Top-view SEM images of (**a-b**) SML100, (**c-d**) SDC16, (**e-f**) SDL43 treated with plasma (Ar, 200 W, 10 s) with and without filter followed by thermal annealing. Micron-scale wrinkled structures are observed from samples plasma treated without the filter (**a, c** and **e**), while perpendicularly aligned microdomains with no surface instability are observed in samples treated with the filtered plasma (**b, d** and **f**).

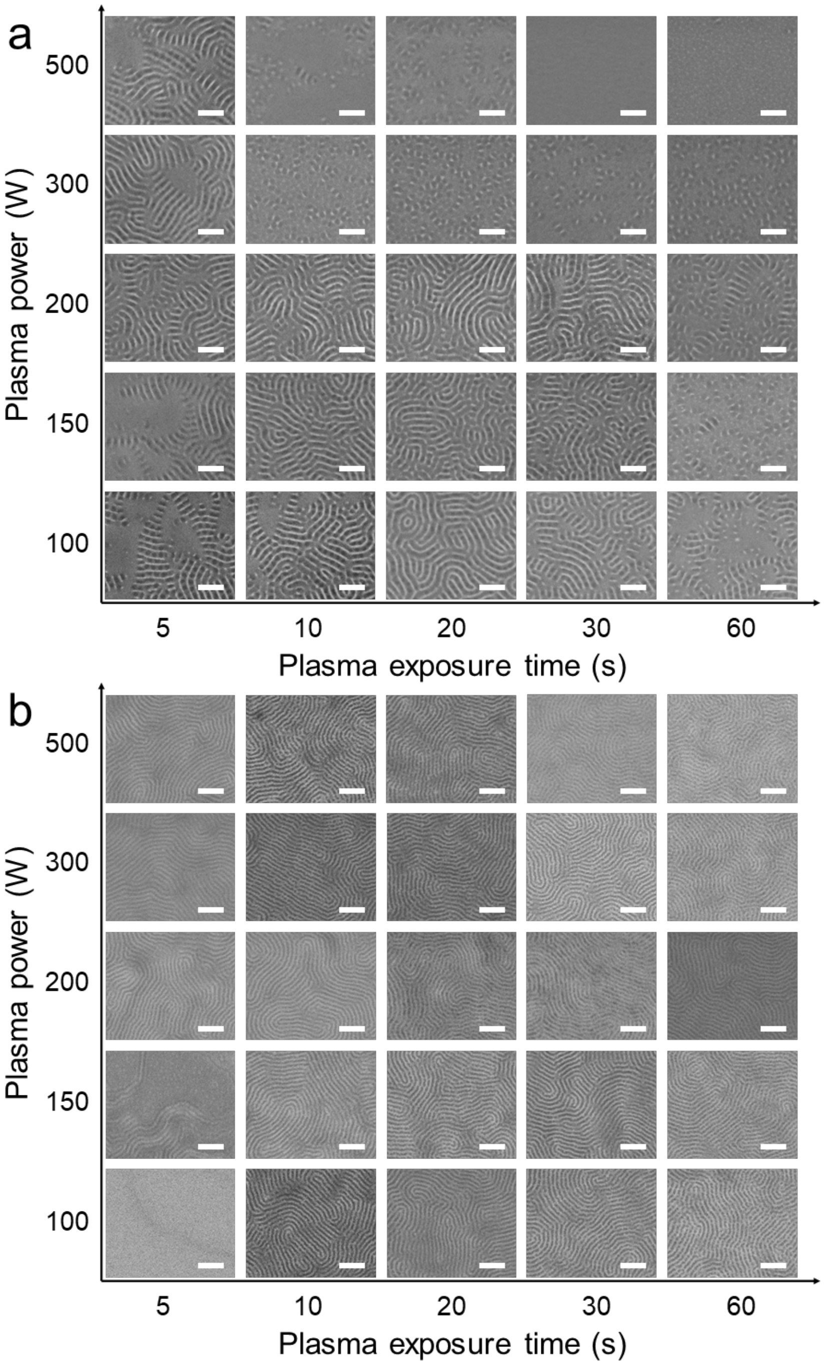

Supplementary Figure 8. Filtered plasma-power and time dependent top surface morphologies. Top surface SEM morphology diagram of filtered Ar plasma treated (a) SML100 and (b) SDL43 films according to plasma power and times. After the thermal annealing at 220 °C for 3 h, additional O_2 plasma etching (90 W, 10 mTorr, 10 s) for SML100 and CF_4 (10 sccm) + O_2 (10 sccm), 450 W, 20 mTorr, 15 s RIE and O_2 RIE (90 W, 10 sccm, 10 s) for SDL43 were carried out to reveal the microdomains.

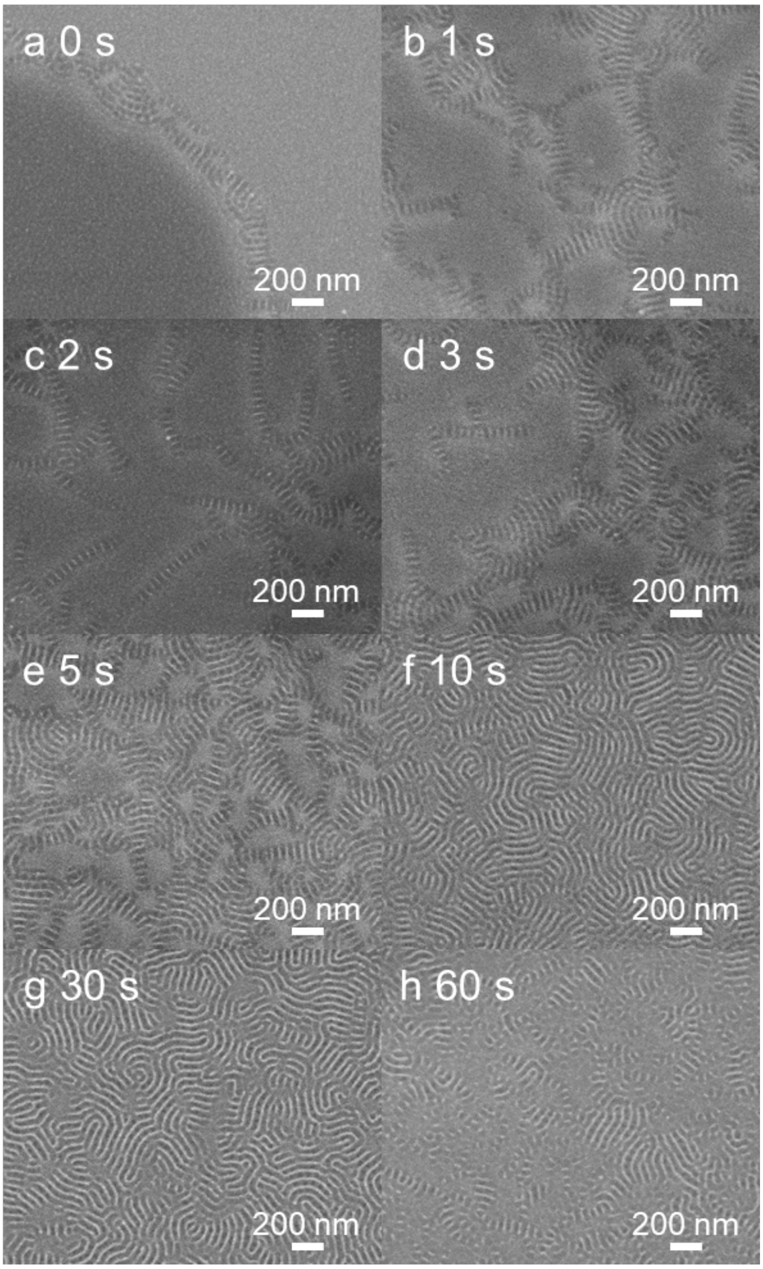

Supplementary Figure 9. Filtered plasma-time dependent top surface morphologies. Top surface SEM morphology diagram of 200 W Ar filtered plasma treated SML100 films with different plasma time from (a) pristine, (b) 1 s, (c) 2 s, (d) 3 s, (e) 5 s, (f) 10 s, (g) 30 s to (h) 60s. After the thermal annealing at 220 °C for 3 h, an O_2 plasma etching step (90 W, 10 mTorr, 10 s) was performed to remove ~5 nm of the film for visualization of the interior perpendicular lamellae.

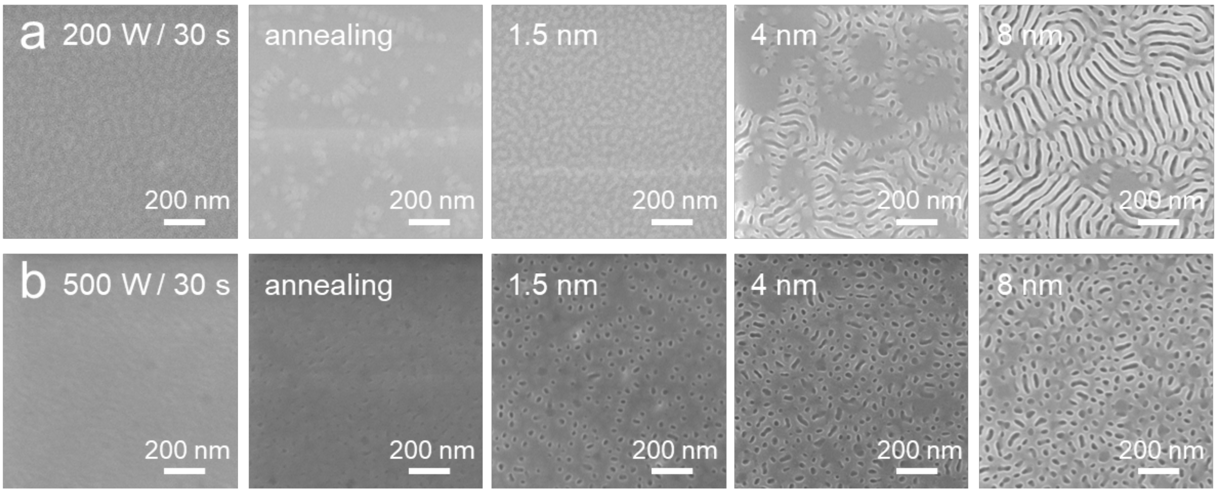

Supplementary Figure 10. Top surface morphology variations according to cover layer etching. Etching reveals the top surface morphology of SML100 treated by a filtered Ar plasma at (a) 200 W and (b) 500 W for 30 s. After the thermal annealing at 220 °C for 3 h, O_2 RIE etching (90 W, 10 sccm) was carried out to remove 1.5 nm, 4 nm and 8 nm of the top surface. Increasing O_2 RIE removed the top layer of the film revealing the perpendicular lamellae.

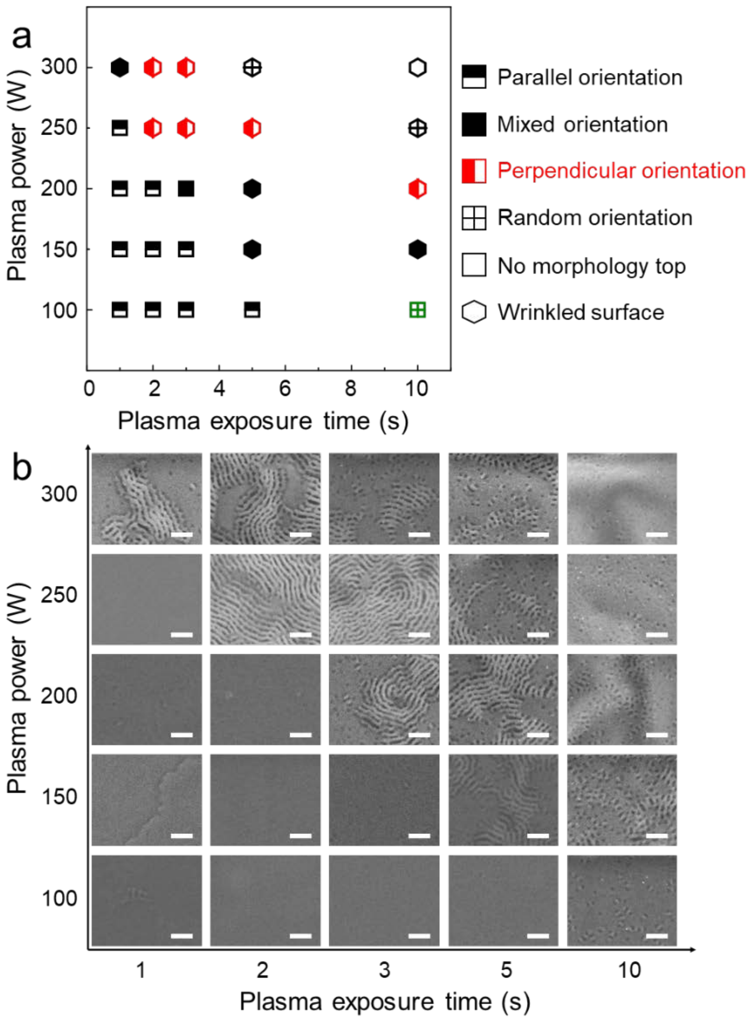

Supplementary Figure 11. Plasma-power and time dependent top surface morphologies without the filter. (a) Orientational phase diagrams and (b) Top surface morphologies of unfiltered Ar plasma treated SML100 films according to plasma power and times. After the thermal annealing at 220 °C for 3 h, additional O_2 RIE (90 W, 10 sccm, 10 s) was carried out to reveal the interior microdomains. The red-colored standing symbols represent conditions that yield the perpendicular orientation of the microdomains, the rectangle represents a flat surface without wrinkles, and the hexagon represents samples with a wrinkled surface. None of the conditions yielded perpendicular microdomains with an unwrinkled surface. A lying rectangle is a parallel orientation, a filled square is a mixed orientation, a filled hexagon is a mixed orientation with wrinkles, a crossed hexagon is a partial perpendicular with wrinkles and an empty hexagon is no morphology on top with wrinkles.

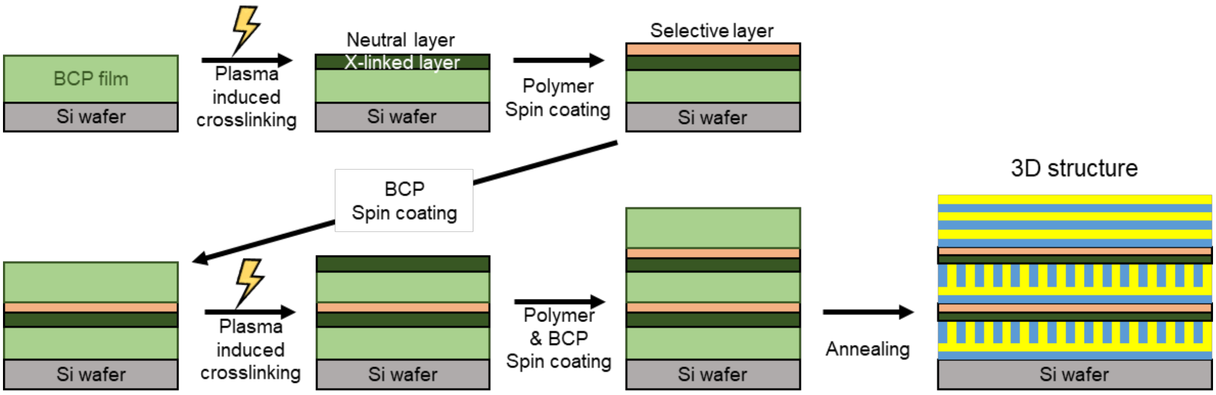

Supplementary Figure 12. Orientation-controlled 3D multilayer fabrications. Schematic images of 3D multilayer fabrication of BCP films with various and independent orientations from the filtered plasma treatment for perpendicular orientation and using a P2VP selective layer (~7 nm) for parallel orientation without any interference between the layers.

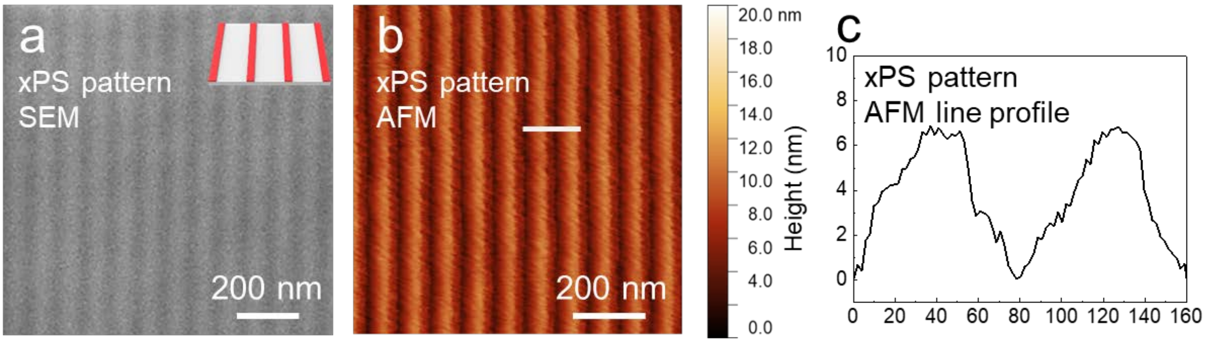

Supplementary Figure 13. Chemically patterned substrate for DSA. (a) SEM, (b) AFM image and (c) topographical line profile of cross-linked PS (xPS) patterns for DSA.

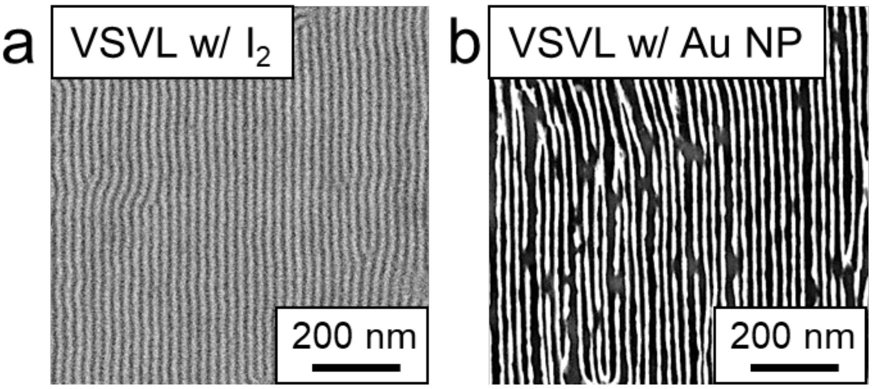

Supplementary Figure 14. Pattern transfer process of VSVL films with filtered plasma. SEM image of (a) I_2 stained VSVL and (b) pattern transferred gold nanowires after removing VSVL with O_2 plasma. VSVL47 films with 46 nm thickness on 84 nm period xPS/PMMA stripes were used to exemplify pattern transfer. The cross-linked top surface of the VSVL47 film was removed by gentle oxygen plasma treatment (90 W for 5 s) and the film was immersed into 5 mmol $HAuCl_4$ / 1 mM HCl solution for 30 min. After removal of polymeric material with oxygen plasma treatment (150 W for 30 s), gold nanowires were obtained.

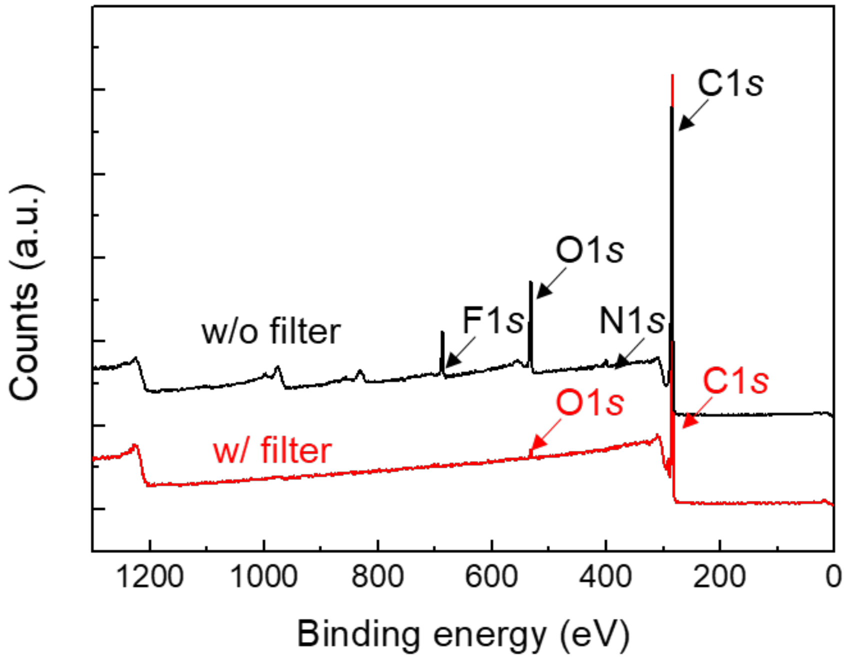

Supplementary Figure 15. XPS data of Ar plasma treated polystyrene w/ (red) and w/o filter (black).

Supplementary References

1. Son, J. G., Bulliard, X., Kang, H., Nealey, P. F. & Char, K. Surfactant-Assisted Orientation of Thin Diblock Copolymer Films. *Adv. Mater.* **20,** 3643–3648 (2008).

2. Zhang, J., Posselt, D., Smilgies, D.-M., Perlich, J., Kyriakos, K., Jaksch, S. and Papadakis C. M. Lamellar Diblock Copolymer Thin Films during Solvent Vapor Annealing Studied by GISAXS: Different Behavior of Parallel and Perpendicular Lamellae. *Macromolecules* **47**, 5711−5718 (2014).
